# TF-finder: A software package for identifying transcription factors involved in biological processes using microarray data and existing knowledge base

**DOI:** 10.1186/1471-2105-11-425

**Published:** 2010-08-12

**Authors:** Xiaoqi Cui, Tong Wang, Huann-Sheng Chen, Victor Busov, Hairong Wei

**Affiliations:** 1 Department of Mathematical Science, Michigan Technological University, 1400 Townsend Drive, Houghton, MI 49931, USA; 2 School of Forest Resources and Environmental Science, Michigan Technological University, 1400 Townsend Drive, Houghton, MI 49931, USA; 3 Biotechnology Research Center, Michigan Technological University, 1400 Townsend Drive, Houghton, MI 49931, USA

## Abstract

**Background:**

Identification of transcription factors (TFs) involved in a biological process is the first step towards a better understanding of the underlying regulatory mechanisms. However, due to the involvement of a large number of genes and complicated interactions in a gene regulatory network (GRN), identification of the TFs involved in a biology process remains to be very challenging. In reality, the recognition of TFs for a given a biological process can be further complicated by the fact that most eukaryotic genomes encode thousands of TFs, which are organized in gene families of various sizes and in many cases with poor sequence conservation except for small conserved domains. This poses a significant challenge for identification of the exact TFs involved or ranking the importance of a set of TFs to a process of interest. Therefore, new methods for recognizing novel TFs are desperately needed. Although a plethora of methods have been developed to infer regulatory genes using microarray data, it is still rare to find the methods that use existing knowledge base in particular the validated genes known to be involved in a process to bait/guide discovery of novel TFs. Such methods can replace the sometimes-arbitrary process of selection of candidate genes for experimental validation and significantly advance our knowledge and understanding of the regulation of a process.

**Results:**

We developed an automated software package called TF-finder for recognizing TFs involved in a biological process using microarray data and existing knowledge base. TF-finder contains two components, adaptive sparse canonical correlation analysis (ASCCA) and enrichment test, for TF recognition. ASCCA uses positive target genes to bait TFS from gene expression data while enrichment test examines the presence of positive TFs in the outcomes from ASCCA. Using microarray data from salt and water stress experiments, we showed TF-finder is very efficient in recognizing many important TFs involved in salt and drought tolerance as evidenced by the rediscovery of those TFs that have been experimentally validated. The efficiency of TF-finder in recognizing novel TFs was further confirmed by a thorough comparison with a method called Intersection of Coexpression (ICE).

**Conclusions:**

TF-finder can be successfully used to infer novel TFs involved a biological process of interest using publicly available gene expression data and known positive genes from existing knowledge bases. The package for TF-finder includes an R script for ASCCA, a Perl controller, and several Perl scripts for parsing intermediate outputs. The package is available upon request (hairong@mtu.edu). The R code for standalone ASCCA is also available.

## Background

Whole-system approaches employing data derived from microarray and high-throughput sequencing technologies require development of new methods for inferring novel knowledge discovery in large-scale data sets. The generation of spatially or temporally interactive transcriptome profiles in a multicellular organism is still challenging and expensive. Therefore methods that can analyze already existing data are urgently needed.

Crop varieties for sustainable biomass production and adaptation to multiple environmental stresses are needed to meet climatic and environmental challenges, and fulfil the world's bioenergy needs. Development of such varieties requires in-depth knowledge of the regulators that play key roles in abiotic stress tolerance and adaptive growth. Understanding the underpinning regulatory mechanisms would enable development of viable solutions to modify plants with augmented stress tolerance and allow sustainable production on marginal lands. Traditional experimental approaches that use candidate gene approaches suffer from biased subjective selection of genes' sets. Thus, often these genes' modifications have little or no impact on the targeted trait and/or in many cases have severe pleitropic effects compromising their commercial deployment. For example, over-expression of DREB1A, and ADR1 results in severely stunted growth [1] and the expression of AtNHX1 negatively impacts many cellular processes including protein transport and modification [2]. Now it is becoming increasingly clear that only systems-based approaches providing thorough knowledge of the intricate genetic networks can provide solutions to these problems and lead to successful translation of biological knowledge into downstream commercial applications [3]. Although our knowledge is incomplete, it has been shown that gene expression is often regulated in a combinatorial manner [4] indicative of the underlying genetic network interactions. Development of methods that can capture these synergistic regulations will provide new insights into the regulatory mechanisms underpinning many biological processes.

Canonical Correlation Analysis (CCA) is a common means to simultaneously analyze the relationships between two sets of variables. However, when applied on large-scale microarray data sets, where the number of genes (variables) greatly exceeds the number of samples, CCA has two major shortcomings: (1) It causes computational problems and inaccurate estimates of parameters; (2) It leads to linear combinations of entire sets of available variables, which may lack biological plausibility and interpretability. To overcome these problems, sparse canonical correlation analysis (SCCA) was recently proposed [5,6]. SCCA, an extension of CCA, can find the maximally correlated relationship between two sets of variables by determining the linear combinations of variables from each set. SCCA provides sparse loadings in the linear combinations and thus results in smaller groups of variables, which can aid the biological interpretability. To further reduce the bias in model selection and number of selected variables, adaptive SCCA (ASCCA) has been recently proposed [5]. ASCCA outperforms SCCA by selecting the correct subset of variables for better discovery of the most plausible model. In addition, ASCCA produces fewer noise variables than SCCA. In this paper, we developed a package, TF-finder that takes advantage of ASCCA to identify TFs involved in a process of interest. As a test case we used TF-finder to identify TFs involved in stress tolerance and adaptive growth. We demonstrated that TF-finder produced interpretable and biologically meaningful data.

We also compared TF-finder with a closely related method, Intersection of Coexpression (ICE) [7], which evaluates a gene from a candidate pool based on how significantly this gene is coexpressed with the number of genes in a positive gene set. We implemented ICE in such a way that the expression data of all TFs were used for identifying novel TFs that are assumed to be involved in the same biological process as these positive TFs that are used as positive gene set. The comparison concluded that TF-finder outperforms ICE in finding novel positive TFs. The novel positive TFs in this study are defined as the newly identified genes that do not belong to the positive TF used as guide genes but are evidenced to be positive genes by present knowledge for involvement in the same biological process.

## Results

We used TF-finder to identify candidate regulatory genes that are involved in salt and drought stress tolerance as well as the adaptive growth under these conditions.

### Identification of salt stress response and tolerance regulators

We applied ASCCA to 109 microarray data sets collected from seven salt stress microarray experiments. The input files contain the expression profiles of 159 positive target genes (non-TFs, Additional file 1) that are known to be involved in salt response and tolerance, 1638 *Arabidopsis *TFs present in Affymetrix ATH1 array, and 13 TFs (AT1G01520, AT2G40950-BZIP17, AT5G39610-ATNAC2, AT5G67450-AZF1, AT3G19580, AT1G52890-ANAC019, AT1G35515-HOS10, AT2G47190-MYB2, AT2G27300-NTL8, AT3G55980-SZF1, AT2G30250-WRKY25, AT2G38470-WRKY33, AT4G28110-MYB41) (Additional File 1) known to be involved in salt response and tolerance. The cluster analysis of the 159 target genes resulted in about 800 clusters that were used to hook TFs in a recursive manner. All TFs identified through this procedure were pooled for frequency calculation. The top 70 genes with highest occurrence frequencies are shown in Additional file 2. Among these genes, 17 TFs were clearly supported by existing evidence to be involved in salt response and tolerance (Additional file 2). For example, WRKY33, AZF2, and NATAC6 were among the list of 13 TFs used as guide genes (Additional file 1). Although the other 14 were all novel, indirect evidence suggests that they are likely involved in this stress response. For instance, CZF1, also known as SZF2, is the most homologous gene to SZF1, and it regulates salt stress responses in *Arabidopsis *[8]. ZAT6 is the most homologous gene to STZ (salt tolerance zinc finger) in *Arabidopsis*. RHL41 (also called ZAT12) is involved in hyperosmotic salinity response [9]. ANAC055 has been found to bind to the early responsive to dehydration (ERD1) stress gene promoter, and over-expression of this gene, together with ANAC019 and ANAC072, causes the expression of several stress-inducible genes that enhance drought tolerance [10]. Over-expression of SZF1 (Salt-inducible zinc finger 1) in transgenic plants caused reduced induction of salt responsive genes and increased tolerance to salt [8]. STZ (salt tolerance zinc finger) was found to increase salt tolerance of calcineurin mutants of wild-type yeast, which appears to be partially dependent on ENA1/PMR2, a P-type ATPase required for Li^+ ^and Na^+ ^efflux. ATAF1 is responsive to wounding and ABA. DREB2A and DREB2B (DRE/CRT-binding protein) are induced upon dehydration and high salinity [11]. ATMYC2 is a positive regulator of ABA signalling. MYBR1 is ABA-regulated and participates in mediating ABA effects [12]. CBF1 functions as a transcriptional activator that binds to the C-repeat/DRE DNA regulatory element in response to low temperature and water deficit [13]. Although CBF1 mainly responds to chilling, the expression of CBF1 also confers salt stress tolerance [14]. BZIP28, an ER-resident TF, serves as a sensor/transducer in *Arabidopsis *to mediate ER stress responses [15].

### Identification of adaptive growth regulators under salt condition

We also used TF-finder to identify TFs controlling growth under the same stress condition. We used the expression profiles of 74 positive target genes that are involved in growth, 10 positive TFs (Additional file 1), and 1640 TFs. 10 positive TFs include AT5G02470-DPA, AT4G16110-ARR2, AT3G13960-GRF5, AT5G53660-GRF7, AT2G16720-MYB7, AT3G49690-MYB84, AT5G20730-NPH4, AT1G13260-RAV1, AT2G33880-HB3, and AT1G32640-MYC2. The resulting top 70 candidate TFs are shown in Additional file 2. Among these genes, 26 TFs have regulatory functions in growth. Three NAC domain-containing TFs: AT3G61910-ANAC066, AT1G60280-ANAC023, and AT5G04400-ANAC077, have been shown to be involved in the differentiation and expansion of petals, stamen, and roots [16-18]. Three closely related basic helix-loop-helix (bHLH) proteins, AT5G53210-SPCH, AT3G06120-MUTE and AT_FAMA, have been identified as positive regulators that direct three consecutive cell-fate decisions during stomatal development [19,20]. AT3G13960-AtGRF5 is one of the nine members of GRF gene family that contain nuclear targeting domain, and is involved in root development [21]. AT2G13570-NF-YB7 encoding LEAFY COTYLEDON1-LIKE is a regulator essential for embryo development [22,23]. KNAT6 is expressed in roots and is required for proper lateral root formation[24]. AT4G27330-SPL plays a central role in patterning of both the proximal-distal and the adaxial-abaxial axes in the ovule and is generally involved in cell differentiation [25]. AT2G35670-FIS2 and AT1G02580-MEA are involved in seed development [26]. CAL is floral homeotic gene encoding a MADS domain protein homologous to AP1 promoting the flower to shoot transformation in ap1 mutants [27]. AT3G15170-CUC1, together wit*h *CUC2 and CUC3, are responsible for shoot organ boundary and meristem formation throughout the different stages of *Arabidopsis *life cycle [28,29]. NUB encodes a protein with a single C(2)H(2) zinc-finger domain and is involved in the growing of later organs [30]. DOT5 is involved in vein patterning, but dot5-1 mutants often have shorter roots, suggesting its functions in root development [31]. INO is involved in ovule development [32]. BLH8 encoded a BEL1 like protein, which was identified to play a role in shoot meristem [33] and ovule development[34]. B3 is differentially expressed in anther, and presumably involved in anther development and differentiation [35]. LBD10 encodes a protein that functions in defining the lateral organ boundaries [36]. AT5G58080-ARR18 encodes a type B response regulator that mediates cytokinins signaling transduction in *Arabidopsis *[37].

### Identification of adaptive growth regulators under drought condition

After showing TF-finder can be used to identify key regulators using data from salt stress experiments, we were interested in extending TF-finder performance testing to a different data set and biological process. We therefore used data from water stress experiments. The three input files contained the profiles of 74 genes involved in various growth processes (Additional file 1), 10 positive TFs (Additional file 1), and all 1640 TFs detected to be expressed in the water stress data set. The top 70 TFs are shown in Additional file 2, among which 21 TFs were previously implicated to be involved in regulation of growth, and one TF, AT2G16720_MYB7, in these 21 genes is a re-discovered positive TF. To avoid spelling out their functions at length, we showed all pieces of evidence that support these genes are positive in Table 1.

**Table 1:** Identified TFs that are involved in growth and stress tolerance under drought condition.

AGI	Category	Gene Symbol	References
AT1G51190	Growth	PLT2 (PLETHORA 2)	[20,46]

AT1G09530	Growth	PIF3 (Phytochrome interacting factor)	[47-49]

AT1G01010	Growth	ANAC001 (NAC domain protein)	[16-18]

AT3G11090	Growth	LBD21 (LOB domain protein)	[36,50]

AT2G36890	Growth	RAX2 (Regulator of axillary meristem)	[51]

AT4G00180	Growth	YAB3 (YABBY3)	[52]

AT5G10510	Growth	AIL6 (Aintegumenta-like)	[53]

AT2G30130	Growth	ASL5; DNA binding	[54]

AT3G24140	Growth	FMA (FAMA)	[19,20]

AT1G02220	Growth	ANAC003 (NAC domain protein)	[16-18]

AT5G02030	Growth	RPL (REPLUMLESS)	[20,46]

AT4G36870	Growth	BLH2 (BEL1-like)	[55]

AT2G24790	Growth	COL3 (CONSTANS-LIKE 3)	[56]

AT2G41070	Growth	EEL (Enhanced em level)	[57,58]

AT3G15030	Growth	TCP4 (TCP family)	[59]

AT3G50750	Growth	BZR1 (Brassinosteroid signalling)	[60]

AT5G44190	Growth	GLK2 (golden2-like)	[61]

AT2G45190	Growth	AFO (Abnormal floral organs)	[62,63]

AT2G01760	Growth	ARR14 (Response regulator)	[64]

AT5G56860	Growth	GNC (GATA, nitrate-inducible)	[65]

AT5G14750	Drought tolerance	MYB66	[66]

AT1G03840	Drought tolerance	MGP (Magpie)	[67]

AT2G40220	Drought tolerance	ABA4 (Insensitive 4)	[68]

AT2G35700	Drought tolerance	ERF38 (ERF family protein 38)	[69]

AT1G13290	Drought tolerance	DOT5 (Defectively organized tributaries)	[31]

AT4G00220	Drought tolerance	JLO (Jagged lateral organs)	[70]

AT1G66370	Drought tolerance	MYB113 (myb domain protein 113)	[71-74]

AT2G38880	Drought tolerance	NF-YB1 (Nuclear factor y, subunit B1)	[75]

AT1G13400	Drought tolerance	NUB (NUBBIN)	[30]

### Identification of regulatory genes from water stress data using ASCCA

To test if TF-finder can identify growth regulators from water stress data, we used three files that contained the profiles of 120 target genes, 9 positive TFs (AT3G57600, AT1G75490, AT5G05410-DREB2A, AT2G47190-MYB2, AT1G54160-NF-YA5, AT2G38880-NF-YB1, AT4G27410-RD26, AT1G69600-ZFHD1, and AT4G28110-MYB41) (Additional file 1) and all 1640 TFs detected to be expressed in the water stress data set. The resulting top 70 genes were found to contain 9 novel TFs (Additional file 2) that are supported by existing evidence to be involved in root growth under water stress condition. Again we are not going to elaborate these genes' functions at length. All pieces of evidence that support these genes are positive were shown in Table 1.

## Discussion

**Figure 1:**
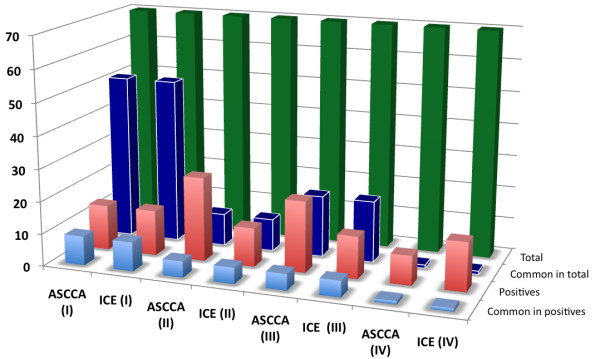
**The efficiency of TF-finder and ICE**. Comparison of TF-finder with ICE in identifying novel TFs involved in: I. salt tolerance in salt stress data; II. growth in salt stress data; III. growth in water stress data; IV. drought tolerance in water stress data. For the color bars (from back to front): green bars represent the top 70 TFs identified, blue bars show the number of common TFs identified by two methods among the top 70, red bars show the number of positive TFs identified by two methods, and the shallow blue are the common positive TFs identified by two methods in the top 70 TFs.

We have developed and shown that the TF-finder package can be used to discover TFs involved in various biological processes. The discovery efficiency varies with both biological processes and genes used to guide the recognition process. To further evaluate the performance of TF-finder, we compared it to the ICE algorithm [7] in identification of TFs involved in namely four biologically processes: (1) salt tolerance, (2) growth under salt stress, (3) growth under water stress, (4) drought tolerance. The inputs for ICE algorithm include the transcriptome profiles of all 1640 TFs and one of the following positive TF sets: 13 TFs involved in salt tolerance, 10 TFs involved in root growth, and 9 TFs involved in water stress tolerance. The results of TFs identified through the two algorithms are shown in Figure 1, and are also listed in Additional file 3

Among the top 70 TFs discovered to be regulators of salt tolerance, 43 are common between the two methods. Among these, 14 novel TFs were identified by both TF-finder and ICE. Among these 14 TFs, 9 were common. This seems to indicate similar efficiency of the two algorithms. However, the comparison between TF-finder and ICE in identifying growth regulators operating during both salt and water stress suggests that TF-finder outperforms ICE. Of the top 70 TFs identified for controlling growth in salt stress, only 10 TFs are common between two methods. 26 and 12 novel TFs identified by TF-finder and ICE respectively were implicated to be positive by the existing annotation with only 5 common. Of the top 70 growth TFs from water stress data, 19 TFs found by two methods were common. Similarly 20 and 13 novel TFs were identified by TF-finder and ICE respectively, and only 5 TFs found by both methods were common. Finally, the efficiency of two methods was compared to discover TFs involved in water stress. Of the identified top 70 TFs, only 1 was common, indicating that despite that both are linear-based methods, TF-finder indeed can identify different TFs. In this case, 9 and 16 TFs were discovered by TF-finder and ICE respectively, and the existing annotation suggests their involvement in response to water stress. This indicates a better performance of ICE in identifying the genes involved in water stress response and tolerance.

The fact that the two methods can recognize different TFs is not surprising because they use different inputs and employ different mechanisms for identifying transcription regulators. Namely, TF-finder hooks TFs using positive target genes. Although both methods use positive TFs, TF-finder uses them as guide genes while ICE as baits to recognize co-expressed genes. Because by design TF-finder identifies a group of TFs controlling a group of targets, it tends to discover combinatorial nature of TFs in regulating a group of target genes. As it is well-known, the drought tolerance gene, proline dehydrogenase in Arabidopsis [38], and GSY2 in yeast [39], as well as ABA-induced gene expression [4] are controlled by a small number of TFs in combinatorial manner. This is mediated by presence of the same stress-responsive *cis*-elements in the promoter sequences of many downstream stress-responsive genes and much less TFs that regulate these genes [40,41]. Therefore we believe that there should be more genes subjected to combinatorial regulation during abiotic stress response and tolerance. In contrast to TF-finder, ICE uses the pair-wise correlation. It thus tends to identify very tightly coupled or co-ordinated TFs by using those that are known to be involved in the same biological process. As the overall efficiency is concerned, in three out of four cases we examined TF-finder identified more TFs for which prior knowledge for involvement in the process of interest existed. The higher efficiency of TF-finder can be ascribed to the repeatedly TF recognition using clustered targets or the use of positive targets, or both of them.

Integration of biological with mathematical models is critically important in discovering novel biological knowledge. However, the complexity of transcription regulation and the lack of data from well-designed experiments impede deriving a biological model using mathematic means. Thus, employment of models (behaviors) of known positive TFs to discover novel TFs is instrumental. We integrated these known TFs for novel TF discovery in a way that the enrichment of these TFs is indicative of a meaningful identification.

In this study, we tried only one set of input files for each case as above-mentioned, namely (1) salt tolerance; (2) growth under salt stress; (3) growth under water stress; and (4) drought tolerance, the number of novel TFs identified is remarkably high. In realty, an even larger number of multiple sets of input files can be formulated to amplify the power of the method. In this regard, the existing TF-finder package can be further improved to take multiple batches of input files, and run iteratively towards more exhaustive results. We believe that such an improvement can lead to the discovery of more novel TFs. In this study, we relied on the existing literature to annotate the identified gene lists and show the efficiency of the TF-finder in identifying positive TFs; however, we indeed noticed there were some highly ranked TFs that were not supported by existing evidence. Due to this reason, we strongly believe there are more positive genes in the identified lists. In a real application, we encourage users to validate those highly ranked but functionally undefined genes by employing experimental means. By the way, we also tested the performance of SCCA [5] on the same data sets with the same inputs. Unfortunately it performed poorly in finding any transcription regulators, which further confirmed the previous conclusion that ASCCA provides better noise filtering and includes fewer uninformative variables than SCCA [5].

With the availability of large volume of gene expression data, and more and more positive target and TF genes being validated by molecular biologists, TF-finder will no doubt have a wide variety of applications in the future. Nevertheless, TF-finder is not useful when these resources are not available, for example, for a newly sequenced species. In addition, TF-finder may not be applicable to some biological processes in which the response of target genes to TF is slow or lagged.

## Conclusions

The integration of existing knowledge base, cluster analysis, and ASCCA algorithm into a package (pipeline) for finding novel TFs with pooled microarray-derived expression data is viable as evidenced by the significant number of discovered TFs. These TFs include previously identified to be involved in mediating abiotic stresses response, indicating that the method can successfully identify TFs involved in the process of interest. In addition, the results imply that combinatorial regulation is dominated in stress response and tolerance, and can be studied through the use of standard positive target (guide) and regulatory genes (bait). Finally, the identification of so many regulatory genes in abiotic stresses is indicative of the involvement of a large complex gene networks. Computational approaches as the one employed by TF-finder can allow insights into the backbone of these genetic networks.

## Methods

**Figure 2:**
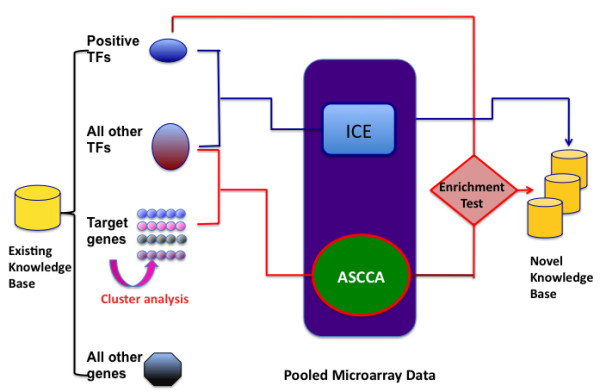
**The workflow of TF-finder package**. Automated package that can recognize transcription regulators controlling a biological process with three inputs: positive TFs, all TFs, and positive targets. ICE: Intersection of Coexpression (ICE) Analysis is integrated into this package for comparison. The package was developed with R, but is called from Perl in Unix /Linux environment. Pre-installation of Eisen's k-means cluster [45] is necessary for auto-clustering analysis.

The workflow of the TF-finder is shown in Figure 2. It covers four methods: k-means clustering, ASCCA, ICE and enrichment test. K-means clustering software was developed earlier [45]. Other methods and the preparation of microarray data are described below:

### ASCCA (Adaptive Sparse Canonical Correlation Analysis)

Assume the expression profiles of all TFs (assume *p *TFs in total) are represented by *X *= {**x**_1_, **x**_2_, ..., **x**_*p*_} with each **x**_*i *_(*i *= 1,..., *p*) being a vector of length *n *(measured on *n *samples), and similarly, the group of target genes is represented by *Y *= {**y**_1_, **y**_2_, ..., **y**_*p*_}with each **y**_*j *_(*j *= 1, .., *q*) being a vector of length *n*. Therefore *X *and *Y *can also be written in the matrix forms:

$$\begin{array}{cc} X={\left(\begin{array}{ccc} x_{11} & \cdots & x_{1p}\\ \vdots & \ddots & \vdots \\ x_{n1} & \cdots & x_{np} \end{array}\right)}_{nxp} & Y= \end{array}{\left(\begin{array}{ccc} y_{11} & \cdots & y_{1q}\\ \vdots & \ddots & \vdots \\ y_{n1} & \cdots & y_{nq} \end{array}\right)}_{nxq}$$

where each **x**_*i *_(*i *= 1, ..., *p*) is a variable in the set *X*, and each **y**_*i *_(*j *= 1, ..., *q*) is a variable in the set *Y*. Then by applying ASCCA on these two sets of data *X *and *Y*, we could get a pair of *p *and *q *entry weight vectors (canonical vectors), **a **and **b**, such that the correlation between the two linear combinations (canonical variates), *X***a **and *Y***b**, is maximized. The canonical vectors **a **and **b **are sparse due to many of their entries being zero, which is achieved by introducing *L*_1 _penalties into the criterion that constrains **a **and **b **(Witten and Tibshirani, 2010). Specifically, in our study, we only focus on the first component, i.e. first pair of canonical vectors, of the ASCCA solution. To facilitate the ASCCA implementation, Parkhomenko et al. (2009) has developed an iterative algorithm as described below (we assume *X *and *Y *are standardized to have columns with zero means and unit variances).

Consider the singular vectors **u **and **v**, which are related with the canonical vectors **a **and **b**by $a=\sum_{XX}^{-1/2}u$ and $b=\sum_{YY}^{-1/2}v$, where Σ_*XX *_and Σ_*YY *_are the variance matrices of *X *and *Y *respectively. Given the penalization parameters, *λ*_*u*_, *λ*_*v *_and *γ*, as well as the initial values **u**^0 ^and **v**^0^, the singular vectors **u **and **v **could be approximated iteratively by the following two steps until convergence:

Step 1 ***Update *u**:

a) ui+1 **u**^*i*+1 ^← *K***v**^*i*^

b) Normalize: **u**^*i*+1 ^← **u**^*i*+1 ^/ ||**u**^*i*+1^||

c) $u_{j}^{i+1}\leftarrow (\left|u_{j}^{i+1}\right|-\frac{1}{2}\lambda_{u}/{\left|u_{j}^{SVD}\right|}^{y})+Sign(u_{j}^{i+1})$ for *j *= 1,2, ..., *p*

where *K *= (*diag*(Σ_*xx*_))^-1/2 ^Σ_*xy *_(*diag*(Σ_*yy*_))^-1/2 ^is a *p *× *q *matrix, Σ_*XY *_is the covariance matrix between *X *and *Y*, *i *is the iteration index and **u**^*SVD *^denotes the first left singular vector (normalized) obtained from a full Singular Value Decomposition (SVD) of *K*. Also,

$$\begin{array}{ccc} {\left(x\right)}_{+}=\{\begin{array}{ccc} x, & \text{if} & x\geq 0\\ 0, & \text{if} & x<0 \end{array} & \text{and} & Sign(x)=\{\begin{array}{ccc} -1, & \text{if} & x<0\\ 1, & \text{if} & x>0\\ 0, & \text{if} & x=0 \end{array} \end{array}$$

d) Normalize: **u**^*i*+1 ^← **u**^*i*+1 ^/ ||**u**^*i*+1^||

Step 2 ***Update *v**:

a) **v**^*i*+1 ^← *K*'**u**^*i*+1^

b) Normalize: **v**^*i*+1 ^← **v**^*i*+1^/ ||**v**^*i*+1^||

c) $v_{j}^{i+1}\leftarrow (\left|v_{j}^{i+1}\right|-\frac{1}{2}\lambda_{v}/{\left|v_{j}^{SVD}\right|}^{y})+Sign(v_{j}^{i+1})$ for *j *= 1,2, ..., *q*

Where **v**^*SVD *^denotes the first right singular vector (normalized) obtained from a full SVD of *K*.

d) Normalize: **v**^*i*+1 ^← **v**^*i*+1 ^/ ||**v**^*i*+1^||

In our analysis, we set the initial values **u**^0 ^and **v**^0 ^as the standardized column and row means of *K*. The penalization parameters *λ*_*u*_, *λ*_*v *_and *γ *are selected by evaluating their different combinations through two-dimensional *k *- fold cross-validation (CV), and then choosing the best combination that maximizes test sample correlation:

$$\Delta_{cor}=\frac{1}{k}\sum_{j=1}^{k}\left|cor(X_{j}{\overset{\wedge }{a}}^{-j},\;Y_{j}{\overset{\wedge }{b}}^{-j})\right|$$

Where X_*j *_and Y_*j *_(the *j*th subset of the *k *- fold CV) are the testing sets, and ${\overset{\wedge }{a}}^{-j}$ and ${\overset{\wedge }{b}}^{-j}$ are the canonical vectors estimated for the training set, in which subset *j *was removed; Since increasing the penalization parameters decreases the number of non-zero terms in **u **and **v**, and for our data **u **and **v **would become zero vectors if *λ*_*u *_and *λ*_*v *_are greater than 0.4. Consequently, we screen the *λ*_*u *_and *λ*_*v *_values from 0 to 0.4 with a step of 0.01, and trace *γ *from 0 to 2 with a step of 0.1. Finally, 35301 (41*41*21) combinations of the three parameters *λ*_*u*_, *λ*_*v *_and γ are examined.

To further evaluate the set of TFs identified by ASCCA, we take the advantage of known positive TFs by examining if the set of identified TFs contains "enough" number of known TFs (Figure 2), which has the similar reasoning as the enrichment test (Rivals et al., 2007) but uses more straightforward and computationally efficient criterion. Denote *N *as the total number of TFs in *X*, *N*_*pos *_as the total number of known positive TFs involved in the same biological process (original input), *N*_*ASCCA *_as the number of TFs fished out by ASCCA, and *N*_*pos*∩*ASCCA *_as the number of known positive TFs that are fished out by ASCCA. Then based on the ratio of positive TFs to total TFs (*N*_*pos *_/ *N*), the expected number of positive TFs identified by ASCCA is (*N*_*pos *_/ *N*)*N_*ASCCA *_which is an ideal criterion to be compared with *N*_*pos*∩*ASCCA*_, the actual number of positive TFs identified by ASCCA. To make the above criterion more stringent such that only the true significant TF sets being retained, we multiply the expected number of positive TFs by an enrichment factor (*EF*) which varies from 1~5. That is, if *N*_*pos*∩*ASCCA *_>EF * (*N*_*pos *_/ *N*)*N_*ASCCA*_, the hooked TF set is saved and discarded otherwise. For all the sample results shown in this study, we set *EF *= 3. In this way, we integrate prior biological knowledge into our mathematical model to deciding if the hooked set of TFs should be retained for further investigation or not.

### Cluster analysis

Before applying ASCCA to extract candidate TFs, we applied k-means clustering method (Eisen et al., 1998) to partition the positive target genes into several clusters (Figure 2) and then use each cluster as an input (*Y*) for ASCCA to bait TFs. The k-means algorithm was selected because: first, target genes in the same cluster are assumed to be co-regulated under the same regulatory machinery and thus each cluster can serve as an ideal bait for ASCCA; second, the result of ASCCA is subject to considerable instability from one input to another, i.e. including or excluding one target gene in *Y *would possibly result in two quite different sets of TFs. This is not surprising because on one hand the sparse canonical vectors (**a **and **b**) are derived from both the greatest correlation between two sets (*X *and *Y*) and correlations among variables within each set; while on the other hand, when there is so much information in the datasets (TFs across whole-genome), there exist several alternative solutions that are almost equally good (Waaijenborg et al., 2008). Consequently, because we aimed to identify TFs by the virtue of their true regulatory causality rather than by chances or due to extraneous factors, we performed ASCCA using many target gene clusters and finally averaged the outcomes to minimize the effect of instability.

Because the optimal number of clusters may not exist since the genes involved in different functional domains are co-regulated in varying sizes, we ran cluster analysis several times by varying the number of clusters from a lower to an upper boundary. At the lower boundary, the average number of target genes in each cluster is 20, and at the upper boundary, the average number of genes in each cluster is 4. For instance, given 100 target genes, k-mean clustering analysis is run 17 times with the average size of clusters varying from 4 to 20, and totally $\sum_{i=4}^{25}n_{i}$ (*n*_*i *_represents the number of clusters when the average size of each cluster is *i*) clusters are processed by ASCCA.

The application of ASCCA on each target gene cluster results in a set of candidate TFs who cooperatively regulate the target genes in this cluster. To extract the truly important TFs from all of the resulting TFs sets, we calculate how many times a TF has been identified by ASCCA. Then the TFs are ranked by the frequency of their occurrence. The more frequent a TF has been identified, the more important is its role in the corresponding biological process. Therefore the list of ranked TFs can provide new hypotheses for further experimental testing. Below is a step-by-step summary of our algorithm:

### Step-by-Step Summary of TF-finder

TF-finder proceeds as follows:

(1) Select a set of positive target genes involved in certain biological process

(2) Select a set of TFs across the genome as input *X*

(3) Set the average size of a cluster *s *= 4

(4) Partition the target genes into *n*_*s *_clusters using k-means clustering method

(5) Use each cluster as input *Y *and apply ASCCA on *X *and *Y*, then save the resulted set of candidate TFs if *N*_*pos*∩*ASCCA *_>EF * (*N*_*pos *_/ *N*)*N_*ASCCA *_and discard otherwise

(6) If *s *< 25, set *s *← *s *+ 1 and repeat steps (3)-(5)

(7) For each TF, calculate the frequency of being captured by ASCCA

(8) Rank TFs by their frequencies following decreasing order

### Comparison of TF-finder with ICE

The principle of ICE is based on "guilty by association". It was implemented in such a way that if a candidate gene is associated with a group of positive genes more often than the others [7], this candidate should be selected. In this study, we used a group of positive TFs to judge if a candidate TF is associated multiple times with the members in this group. Due to the presence of multigenic regulation in the gene network, it is usual that transcription regulators controlling the same set of target genes are coordinated or co-expressed. Therefore, we employed Spearman correlation to 'associate' a candidate TF to a number of positive TF. Detail for ICE implementation is described as following. Let *Y *= {*y*_1_,*y*_2_, ..., *y*_*m*_} is a set of known positive TFs controlling a biological process, and *X *= {*x*_1_,*x*_2_, ..., *X*_*n*_} is a set of TFs across genome with *X*∩*Y *= *ϕ*. A Spearman rank correlation *ρ*_*ij *_is calculated between any pair of *x*_*i *_and *y*_*j *_(*i *= 1, ..., *n*, *j *= 1, ..., *m*), and *X*_*i *_and *y*_*j *_are considered linked when *ρ*_*ij *_is larger than a pre-specified threshold ρ_0_. In our study, we set *ρ*_0 _= 0.6. Then all TFs in *X *are sorted by the number of links to *Y*. The genes at the top of the list have more links to *Y*, and thus are the candidate regulating genes involved in the biological process. Since each selected *x*_*i *_is located at the "intersection" of multiple elements from *Y *in a network, we termed this approach as "the intersection of coexpression (ICE)".

### Preparation of microarray data sets

Microarray data sets were downloaded from multiple resources. Salt stress experimental data set contains108 chips from 6 experiments (GSE7636, 7639, 7641, 7642, 8787, 5623) and was downloaded from NCBI GEO http://www.ncbi.nlm.nih.gov/geo/. Water stress data sets were downloaded from European Arabidopsis Stock Centre's website http://arabidopsis.info/ and include 62 chips from 3 experiments of AtGenExpress: Stress Treatments (Drought stress) contributed by AtGenExpress Consortium. All data mentioned above are derived from hybridization of Affymetrix 25 k ATH1 microarrays [42]. The original CEL files were processed by the robust multiarray analysis (RMA) [43] algorithm using the Bioconductor package. For quality control we used methods that were previously described [44]

### Availability of software package

The ASCCA package was written in R. A wrapper for calling ASCCA, and a number of parsers were written in Perl. To facilitate use of this package, we release for public use the original codes rather than executables. The users need to use an Unix/Linux environment where R and Eisen's k-means clustering package [45] are installed. Installation of Perl is not necessary because it is usually carried by the Linux/Unix operating system. Interested users can receive the package by sending email to: hairong@mtu.edu.

## Authors' contributions

XC implemented ASCCA in R, and wrote the method part of manuscript. TW prepared the microarray data. HSC directed XC for implementing ASCCA, and revised manuscript. VB supported the project and wrote some parts of manuscript. HW automated the package, ran the TF-finder to produce the results, and wrote the manuscript. All authors read and approved the final manuscript.

## Acknowledgements

This project was supported by Agriculture and Food Research Initiative Competitive Grant no. 2009-65504-05767 from Agriculture Plant Feedstock Genomics for Bioenergy: A Joint Research Program of USDA and DOE Program

## Supplementary Material

Additional file 1 **Positive target genes and positive TFs used for testing TF-finder**. This is a Microsoft Excel file (.xls) that can be visualized using the Excel contained in Microsoft Office package. Click here for file

Additional file 2 **Novel TFs recognized by TF-finder**. This is a Microsoft Excel file (.xls) that can be visualized using the Excel contained in Microsoft Office package. Click here for file

Additional file 3 **Comparison of TFs recognized by TF-finder and ICE**. This is a Microsoft Excel file (.xls) that can be visualized using the Excel contained in Microsoft Office package. Click here for file
